# Enhanced Remediation of Phenanthrene and Naphthalene by Corn-Bacterial Consortium in Contaminated Soil

**DOI:** 10.3390/plants13202839

**Published:** 2024-10-10

**Authors:** Lu Gao, Charles Obinwanne Okoye, Congsheng Wang, Feiyue Lou, Jianxiong Jiang

**Affiliations:** 1 Biofuels Institute, School of Emergency Management, School of Environment and Safety Engineering, Jiangsu University, Zhenjiang 212013, China; lgao@ujs.edu.cn (L.G.); charles.okoye@unn.edu.ng (C.O.O.); wangcongsheng1988@163.com (C.W.); loufeiyao@163.com (F.L.); 2 School of Life Sciences, Jiangsu University, Zhenjiang 212013, China; 3 Department of Zoology & Environmental Biology, University of Nigeria, Nsukka 410001, Nigeria

**Keywords:** phenanthrene, naphthalene, soil bacteria, corn plants, removal efficiency, biomass yield

## Abstract

The persistent and hazardous nature of polycyclic aromatic hydrocarbons (PAHs) released into the soil has become a critical global concern, contributing to environmental pollution. In this study, the removal efficiency of phenanthrene and naphthalene degradation by complex flora or pure bacteria combined with corn and their effects on the growth of corn, pH, and the number of soil bacteria were investigated using a pot experiment. The results indicate that the corn remediation method (P) outperformed degrading bacteria remediation (B) for phenanthrene, yet the combination (PB) exhibited significantly higher removal efficiency. The degradation efficiency of PB methods increased over time, ranging from 58.40% to 75.13% after 30 days. Naphthalene removal showed a similar trend. Soil pH, influenced by remediation methods, experienced slight but non-significant increases. The number of degrading bacteria increased with combined methods, notably with PB-W1 and PB-W2 treatments. Corn accumulated phenanthrene and naphthalene, with higher concentrations in roots. Remediation by the combined corn and degrading bacteria slightly increased PAH accumulation, indicating potential root protection. Biomass yield analysis revealed the inhibitory effects of PAHs on corn growth, decreased by degrading bacteria. PB-W1 and PB-EF3 demonstrated the highest fresh weight and moisture content for stem and leaf biomass, while PB-F2-6 excelled in root biomass. Overall, combined remediation methods proved more effective, which underscores the potential of the corn and degrading bacteria consortium for efficient PAH remediation in contaminated soil.

## 1. Introduction

Polycyclic aromatic hydrocarbons (PAHs) are extensively researched organic pollutants commonly discovered in abandoned industrial zones [1]. The United States Environmental Protection Agency (US EPA) classified the majority of PAHs, such as benzo[a]anthracene, benzo[g,h,i]perylene, benzo[k]fluoranthene, indeno[1,2,3-c,d]pyrene, dibenzo[a,h]anthracene, benzo[b]fluoranthene, benzo[a]pyrene, anthracene, fluoranthene, chrysene, fluorene, acenaphthylene, acenaphthene, pyrene, phenanthrene, and naphthalene, as environmental contaminants of concern based on their abundance and toxicity [2,3]. These compounds, characterized by two or more fused benzene rings, exhibit diverse structures and toxicity levels. Their environmental persistence poses a significant health risk as they can enter the food chain, leading to bioaccumulation and biomagnification [4]. In soil, PAHs display low mobility and high durability, and their presence is influenced by factors such as temperature, pH, soil organic matter content, and the historical contamination of the soil [5]. Soil contamination by PAHs has become a significant environmental concern due to their persistence, toxicity, and adverse effects on human health and ecosystems [4]. Because they are hydrophobic, these PAHs attach themselves to soil and sediment particles, decreasing their availability for biological metabolism [6]. According to the results of a national soil survey in 2014, PAHs have become one of the main forms of organic pollutants in the farmland in China [7]. Toxic PAHs can accumulate in crops and enter the human body along the food chain, posing a threat to human health. Remediation of PAH-contaminated soil is crucial to mitigating these risks and restoring the ecological balance [8]. Therefore, research on the enhanced degradation of PAHs is one of the critical approaches to solving petroleum-related soil pollution.

Traditional remediation methods often involve physical or chemical treatments, which can be expensive, environmentally intrusive, and may lead to secondary pollution [9]. Bioremediation, a technique that uses biological agents such as microorganisms and plants that are highly tolerant to the toxicity of hydrocarbons, has recently attracted much attention as an economical and ecologically beneficial way to remediate contaminated environments [10]. The first generation of phytoremediation technology uses hyperaccumulators and has obtained positive effects on restoration and ecology. However, these technologies are challenging to apply on contaminated farmland due to the lack of economic output for the farmers. Phytoremediation, mainly using crops, has gained significant attention for remediating contaminated farmland [11]. Among these crops, corn is a low accumulator of PAHs compared to wheat, carrots, and soybeans [12,13]. Despite its robust root system and adaptability to diverse soil conditions, which makes it a promising candidate for soil PAH remediation [14], soil microorganisms have also been shown to improve the effectiveness of phytoremediation in polluted soil by reducing the phytotoxic effects of PAHs and facilitating their absorption by plants [15]. Therefore, introducing PAH-degrading microorganisms offers a synergistic approach to degrading soil organic pollutants [16]. In PAH-contaminated environments, where only certain microorganisms can survive, microbial equilibrium is disrupted, and the inoculation of appropriate microbial strains emerges as a viable solution for soil restoration [17]. Recent studies have utilized fungal isolates and bacterial strains for remediating PAH-contaminated soils [18,19].

Although some degrading bacteria and plant species were investigated recently to determine their ability to degrade individual PAHs in diverse environments [20], comprehensive information on the biodegradation of these strains and their combination with plants on complex PAHs is still scarce. Furthermore, the complex interplay between the plant–microbe–soil system in the context of PAH remediation remains insufficiently elucidated, necessitating a comprehensive investigation that assesses the effectiveness of such remediation strategies under laboratory conditions.

Based on the preceding information, this study aims to evaluate the remediation efficiency of corn plants alone and their combination with specific strains of degrading bacteria for PAH-contaminated soil. A preliminary investigation showed that the selected bacteria and corn plants exhibit diverse capabilities for degrading phenanthrene and naphthalene [19,21]. By assessing various remediation methods for contaminated farmland, this study could provide valuable insights into the feasibility and effectiveness of corn and combined bacterial remediation strategies for PAH-contaminated soil in agricultural areas.

## 2. Results

### 2.1. Removal Rates of Phenanthrene and Naphthalene in the Contaminated Soil

**Figure 1:**
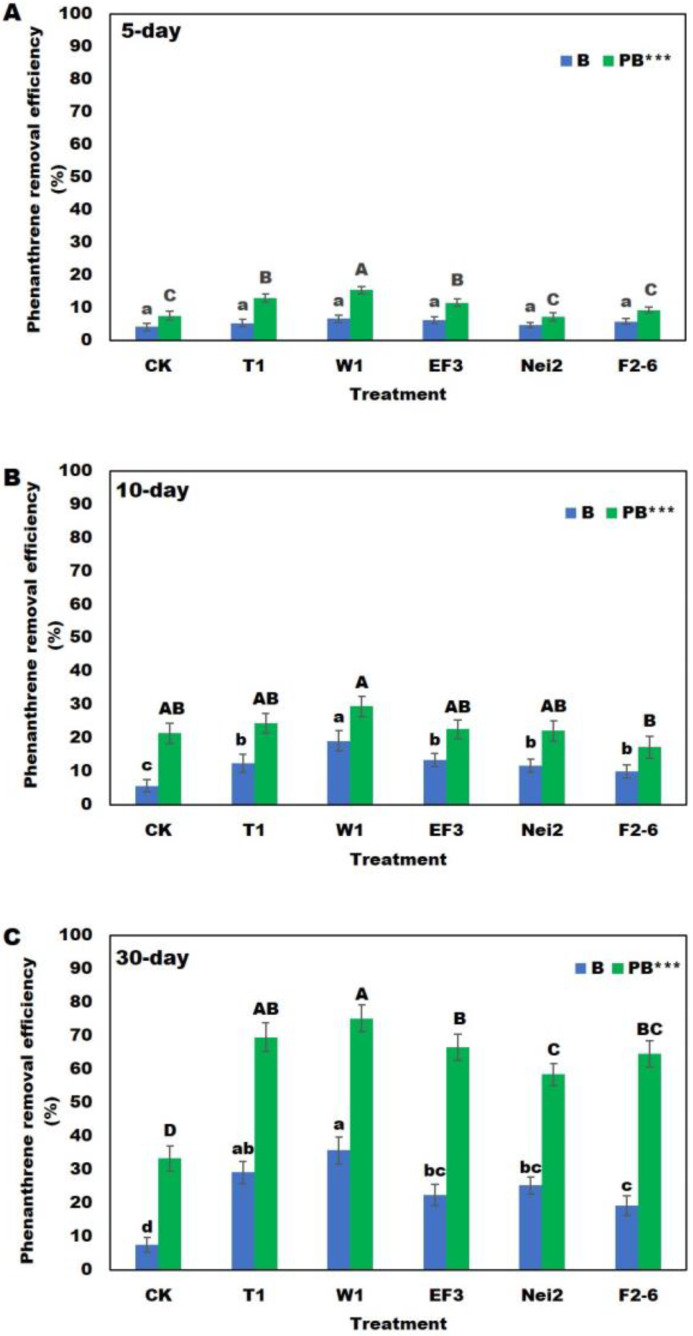
Removal efficiency of phenanthrene in the soil after (**A**) 5; (**B**) 10; and (**C**) 30 degradation days. B, contaminated soil treated with degrading bacteria; PB, contaminated soil treated with degrading bacteria and plants. Different lowercase and uppercase letters indicate a significant difference in phenanthrene removal efficiency (*p* < 0.05) in the B and PB groups, respectively; *** indicates a significant difference between the B and BP groups (*p* < 0.001).

The results showed that phenanthrene removal efficiency by the corn remediation method (P) (7.48%, 21.45%, and 33.28% for 5, 10, and 30 days, respectively) was better than that of the degrading bacteria remediation method (B) (4.65–6.54% for 5 days, 10.36–19.13% for 10 days, and 19.16–35.66% for 30 days). However, the removal efficiencies of all the corn and degrading bacteria remediation methods (PB) (7.17–15.30% for 5 days, 17.27–29.45 for 10 days, and 58.4–69.55% for 30 days) were significantly higher than those of the P or B remediation methods. Also, with the continuous extension of the cultivation time, the degradation efficiencies of PB remediation methods became more apparent (Figure 1). After 30 days, the average removal efficiency of each PB (PB-T1, PB-W1, PB-EF3, PB-Nei2, and PB-F2-6) on phenanthrene in soil ranged from 58.40% to 75.13%. Of those, the highest removal efficiency was obtained by the PB-W1 remediation method (75.13%), followed by PB-T1 (69.55%), while the lowest was obtained by the PB-F2-6 remediation method (58.40%).

**Figure 2:**
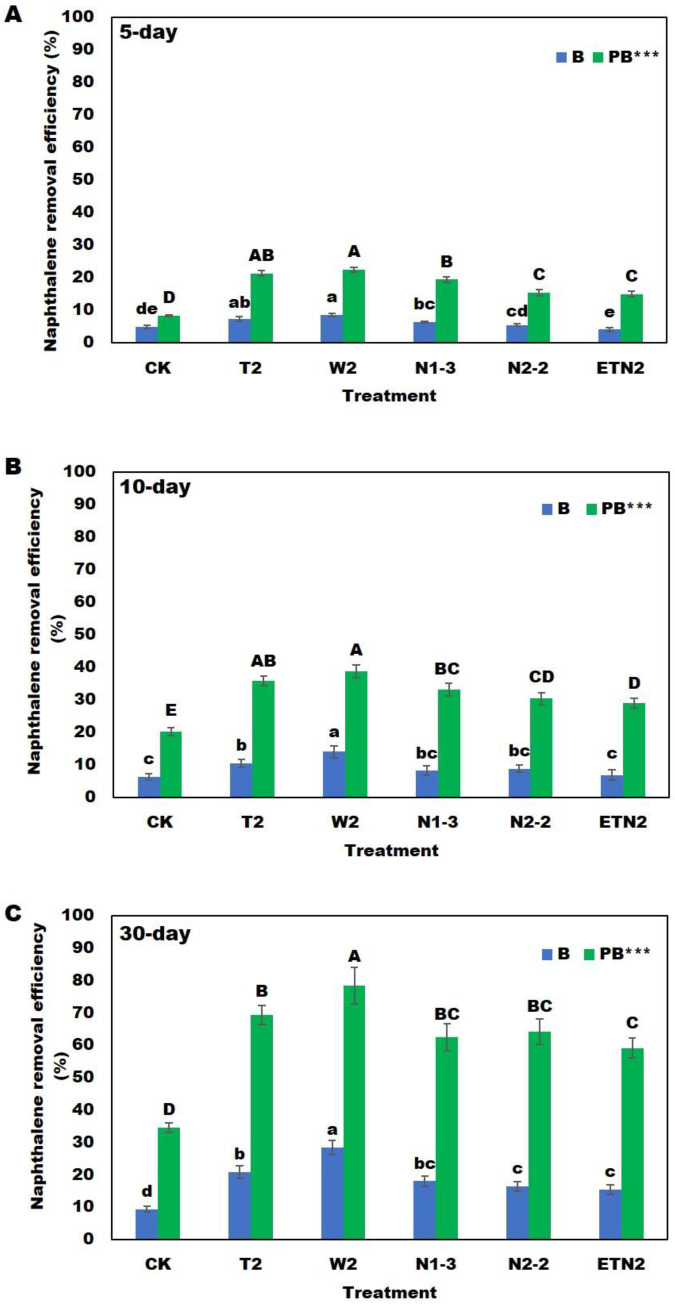
Removal efficiencies of naphthalene in the soil after (**A**) 5; (**B**) 10; and (**C**) 30 degradation days. B, contaminated soil treated with degrading bacteria; PB, contaminated soil treated with degrading bacteria and plants. Different lowercase and uppercase letters indicate significant differences in phenanthrene removal efficiency (*p* < 0.05) in the B and PB groups, respectively; *** indicates a significant difference between the B and BP groups (*p* < 0.001).

For the naphthalene degradation in soil, the results showed that the varying trend was very similar to that of phenanthrene. The removal efficiencies of naphthalene by all the PB remediation methods (15.02–22.50% for 5 days, 29.00–38.73% for 10 days, and 59.19–78.55% for 30 days) were significantly higher than those of the P (8.38%, 20.13%, and 34.55% for 5, 10, and 30 days, respectively) or B (4.12–8.61% for 5 days, 6.90–14.01% for 10 days, and 15.43–28.41% for 30 days) remediation methods. Moreover, the removal efficiencies of naphthalene by the two combined methods of PB-W2 (22.50%, 38.73%, and 78.55% for 5, 10, and 30 days, respectively) and PB-T2 (21.39%, 35.85%, and 68.40% for 5, 10, and 30 days, respectively) were significantly higher than those of other PB methods of corn and pure bacteria (150.2–19.67% for 5 days, 23.00–33.24% for 10 days, and 59.19–64.17% for 30 days) (Figure 2). After 30 days, the average removal efficiencies of various PB remediation methods for naphthalene in soil ranged from 59.19% to 78.55%. The highest removal efficiency was found for the PB-W2 remediation method (78.55%), while the lowest was found for the PB-ETN2 combined remediation method (59.19%).

### 2.2. Effect of Potting Soil pH on Phenanthrene and Naphthalene Removal Efficiency

**Figure 3:**
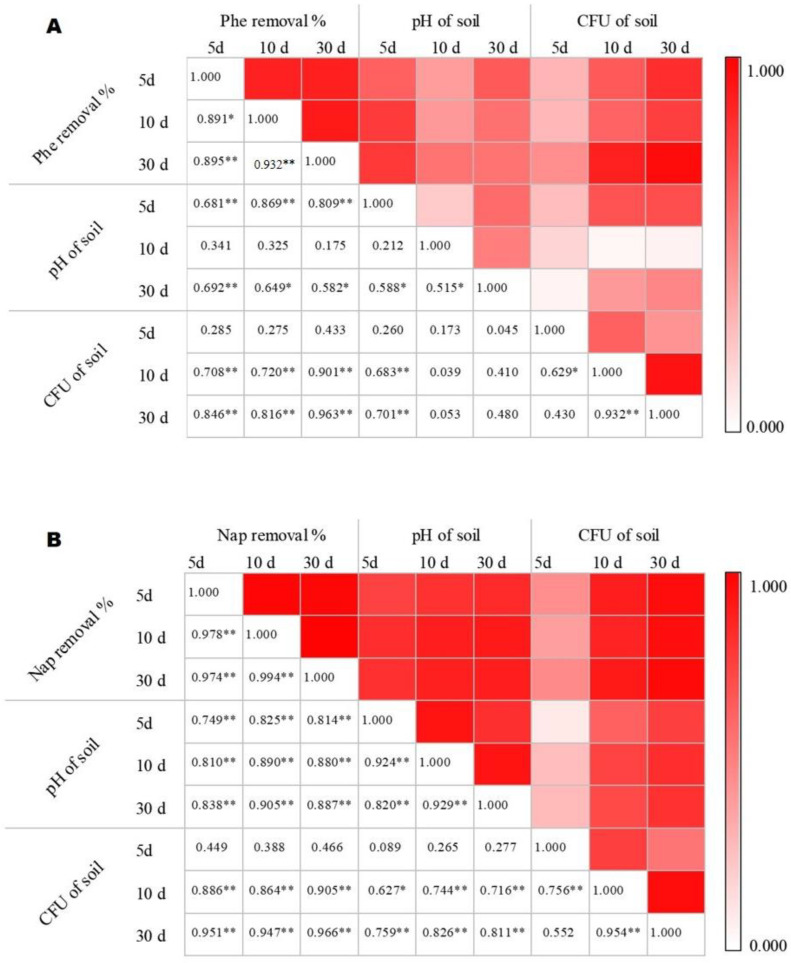
Pearson’s correlation analysis among the PAHs removal efficiency, pH of the soil, and amount of degrading bacteria (10^8^ CFU·g^−1^) in potting soil. (**A**) Phenanthrene-contaminated soils; (**B**) naphthalene-contaminated soils. * and ** indicate significant correlation at *p* < 0.05 and *p* < 0.01 levels (bilateral).

This study determined the pH value of potting soil using different remediation methods after contamination with phenanthrene and naphthalene. The results showed that, with the extension of the test time, the soil pH value slightly increased among the different remediation methods and CK (Table S3). After 5- and 30-day treatments of phenanthrene and 5-, 10-, and 30-day treatments of naphthalene, there were significant positive correlations between the potting soil pH and the removal efficiency of the corresponding pollutions (Figure 3). The pH of potting soil, which was affected by degrading bacteria, played an important role in the removal of phenanthrene or naphthalene from contaminated soil.

### 2.3. Effect of Rhizosphere Bacteria Number on Phenanthrene and Naphthalene Removal Efficiency

The number of rhizosphere bacteria in the soil showed a consistent trend (Table S4). After 10 and 30 days of treatment, there was a significant positive correlation between the number of rhizosphere bacteria and the removal efficiency of phenanthrene and naphthalene (Figure 3). On the other hand, compared to CK, the contaminated soil treated with degrading bacteria showed a gradually decreasing trend in the number of bacteria with time. In contrast, in all the soils treated with BP methods, the number of soil bacteria increased to the highest (*p* < 0.05) numbers, 11.83–13.83 and 12.00–13.67 times, respectively, those of phenanthrene- and naphthalene-contaminated soils. The increase in the number of rhizosphere microorganisms caused by the addition of degrading bacteria is one of the key factors for the rise in soil phenanthrene and naphthalene removal rates, and planting plants such as corn plays a crucial role in promoting the colonization of degrading bacteria.

### 2.4. Accumulation of Phenanthrene and Naphthalene in Corn

This study quantified the levels of phenanthrene or naphthalene in the shoots and roots of the corn plants to evaluate the effects of introducing degrading bacteria on the accumulation of these contaminants in corn. Overall, the average accumulation of phenanthrene in the shoots of corn per kilogram of fresh weight was 0.063–0.131 mg·kg^−1^, and the average accumulation of phenanthrene in the underground roots per kilogram of fresh weight was 0.215–0.380 mg·kg^−1^ (Table 1). The accumulation of naphthalene in the shoots was 0.103–0.153 mg·kg^−1^, and the accumulation of naphthalene in the roots was 0.343–0.427 mg·kg^−1^ (Table 1). Furthermore, the TF showed no significant difference, ranging from 0.294 to 0.348 and from 0.274 to 0.358 for phenanthrene and naphthalene, respectively. In addition, the content of phenanthrene in the shoots and roots of corn plants under the BP remediation method was significantly higher than that of the P remediation methods. At the same time, there was no significant difference in naphthalene accumulation in shoots and roots among the remediation methods.

### 2.5. Effects of Inoculating Degrading Bacteria on Corn Biomass Yield

The performance of the PB methods for phenanthrene and naphthalene in the corn biomass yield was further evaluated to reveal the effectiveness of applying degrading bacteria to alleviate the toxic effects of PAHs. Overall, the shoots and roots of corn under different treatments showed that, after 30 days, 100 mg·kg^−1^ of phenanthrene or naphthalene in the soil had a strong inhibitory effect on the growth of corn plants, and the biomass of shoots and roots significantly decreased. In the treatment with the application of phenanthrene-degrading bacteria, the average fresh weight of the shoots was 4.16–5.32 g, and the average fresh weight of roots was 1.78–2.54 g, both of which increased in the BP remediation methods compared to P, with the CP having the highest fresh weight (5.32 g) in shoot biomass (Table 2). However, among the combined corn and degrading bacteria remediation methods, PB-W1 (5.24 g) and PB-EF3 (83.71%) had the highest fresh weight and water content for shoot biomass. At the same time, PB-F2-6 had the highest fresh weight (2.54 g) and water content (78.35%) for root biomass.

After 30 days of potted cultivation, the fresh weight of the shoot biomass of a single plant of corn with the addition of naphthalene-degrading bacteria was 3.80–5.61 g, and the fresh weight of root biomass was 1.68–2.31 g, with the CP having the highest fresh weight (2.31 g) and moisture content (77.31%) in root biomass (Table 2). The corn biomass yield of all BP remediation methods was significantly higher than P (*p* < 0.05) and recovered to the CP level. Moreover, the root biomass of PB-W2 and PB-ETN2 had the highest fresh weight (5.61 g) and water content (83.37%) for shoot biomass.

## 3. Discussion

The present study revealed the combined corn and degrading bacteria remediation method’s performance in significantly removing phenanthrene and naphthalene compared to the corn or degrading bacteria remediation methods, reaching 75.13% and 78.55% after 30 days. This is similar to the experimental results of the combined remediation of phenanthrene, naphthalene, and other PAHs in soil by *Festuca arundinacea* inoculated with *Bacillus licheniformis* and *Bacillus mojavensis* isolated from oil-contaminated soils by Eskandary et al. [22], in which it was reported that the removal percentages of phenanthrene by the combined remediation method were higher (*p* < 0.05) than *Festuca* alone. According to Zuzolo et al. [23], the plant–microorganisms association, arising from the inoculation of consortia, is a rising remediation technology to effectively improve the degradation efficiency of PAHs in soil. The authors reported that *Verbascum sinuatum* L. was utilized in conjunction with a microbial consortium comprising fungi and bacteria across three distinctively contaminated soils. After 240 days, the highest removal of PAHs reached up to 68%. Phenanthrene and naphthalene contaminants in soil can be partially dissolved naturally through leaching and volatilization, which accounts for a relatively small amount and is referred to as abiotic loss. The remediation of PAHs by degrading bacteria is due to abiotic loss and metabolic activities of degrading bacteria in soil [2], while the removal efficiency of the latter is much higher than that of the former. Numerous bacteria have been identified for their ability to degrade PAHs through either metabolic processes or co-metabolism. Aligning with findings from our previous study [19], the combinations of two or more bacteria showed tremendous degradation of low-molecular-weight PAHs, like phenanthrene, pyrene, and naphthalene. In this study, the complex degrading flora, like W1 and W, showed significantly higher efficiency in removing phenanthrene and naphthalene in both the B and BP group remediation methods.

Although the role of degrading bacteria was very significant in the present study, the results also indicated that corn crops played an undeniable core role in phenanthrene and naphthalene bioremediation. The biodegradation rates of PAHs are highly variable and depend not only on PAH structure, but also on physicochemical parameters such as the soil pH [24,25]. In this study, soil pH slightly increased with the remediation process among the different remediation methods for phenanthrene- and naphthalene-contaminated soils. Meanwhile, the PB group remediation methods significantly affected the increase in soil pH levels (PB-EF3 = 7.77 and PB-ETN2 = 7.71) after 30 days. Pawar [26] reported a soil pH of 7.5, which also increased the bacterial population. In our study, pH emerged as a crucial factor influencing the performance of degrading bacteria. This aligns with earlier findings that highlight pH’s ability to modify the solubility of organic carbons, impact microbial metabolic processes, and enhance bioavailability [12]. Corn may play a role in regulating the pH value of contaminated soil, thereby affecting the removal efficiency of phenanthrene and naphthalene by degrading bacteria.

The harmful impacts of PAHs are influenced not just by the physicochemical characteristics of the contaminant or the inherent tolerance of the plant, but also by the ability of natural microbial populations to degrade PAHs. Previous studies have found that the removal efficiency of pollutants is affected by the composition and quantity of bacteria [19]. Additionally, the plant’s capacity to stimulate indigenous soil microbes for contaminant degradation plays a significant role [27]. Plants provide nutrients, substrates, and signaling molecules to promote the colonization of degrading microbes via root exudates. In turn, through the plant–microbe co-metabolic network, the degrading bacteria promote growth and enhance plants’ tolerance. With the development of next-generation sequencing, the interaction mechanism between rhizosphere-degrading bacteria and plants is under intensive study via multiomics techniques, including metagenomics, metatranscriptomics, and metaproteomics [19,28,29].

The combined remediation methods for corn and degrading bacteria showed a significantly higher trend in the number of soil bacteria. In addition to providing the rhizosphere environment to promote degrading bacteria colonization and improve degradation ability, plants are also an important component of bioremediation technology [30]. Plants can transform the polluted waste into biomass, carbon dioxide, and certain new compounds with lower toxicity through absorption and internal metabolism [14]. The present results consistently showed that the accumulation of phenanthrene and naphthalene in the underground roots and aboveground stems and leaves of corn plants under the BP remediation method was slightly higher than that of the control plants without degrading bacteria. Furthermore, corn roots accumulated significantly more PAHs than the shoots. Yu et al. [31] reported a higher accumulation of PAHs in the roots of ryegrass. Also, the accumulation of phenanthrene or naphthalene in corn roots is usually 3 to 4 times that in the upper stems and leaves of corn fields, which is consistent with the characteristics of absorption by corn roots and transportation to the stems and leaves [32]. The accumulation results of phenanthrene and naphthalene in corn plants showed that the contents of phenanthrene and naphthalene in corn plants slightly increased after adding bacteria, which may have been because the presence of degrading bacteria alleviated the damage of phenanthrene and naphthalene toxins to the roots. The root biomass increased, which was consistent with the results of root biomass measurement. According to Gao et al. [33], PAHs such as phenanthrene and naphthalene seem much more easily taken up and translocated into plant roots via hyphae. PAHs can infiltrate plant surfaces and travel through the intracellular space and vascular system.

Nevertheless, the interactions between corn and PAHs-degrading bacteria on translocations of PAHs in plant tissues are not yet thoroughly understood. Bioremediation not only aids in removing pollutants from the soil, but also provides potential solutions for reducing health risks in contaminated environments. In agricultural settings, crops that have accumulated excessive PAHs from the soil may be classified as carcinogenic due to the risk of human ingestion and absorption through them [34,35]. However, the corn used in this study was reported as a low-accumulate crop of PAHs [12,13].

The accumulation of polycyclic aromatic hydrocarbons (PAHs) in both shoots and roots was lower in crops compared to other plants used in 100 Mm PAH-contaminated soils [32,36,37]. Also, the intensification of phenanthrene and naphthalene accumulation in corn was not observed due to the increased bioavailability of pollutants resulting from the activation of degrading bacteria in this study. The combination of degrading bacteria with the corn bioremediation method shows potential feasibility in restoring farmland contaminated with PAHs, especially in cases of low to moderate pollution levels. Chen et al. [38] reported a moderate carcinogenic risk, signifying a widespread exposure risk to farmland soil PAHs among residents. Therefore, understanding the mechanisms of PAH accumulation and implementing strategies for efficient bioremediation can be crucial in minimizing these risks. Furthermore, establishing a limit threshold for food crops and rigorous monitoring should be warranted to avoid the risk of cancer for humans in agricultural areas and farmlands [34,39].

PAHs beyond certain thresholds adversely impact both plant germination and subsequent growth, as well as the overall biomass yield [27]. In this study, shoot and root biomass yield and corn were reduced by the presence of phenanthrene and naphthalene in the contaminated soil. The degrading bacteria alleviated the inhibitory effect of pollutants on corn growth, especially significantly on shoot biomass. The study concluded that the uptake of phenanthrene by plant roots involves an active, carrier-mediated process that consumes energy [40]. The addition of different degrading bacteria does not affect the absorption of fertilizer by plants and the growth of plants. Microorganisms can decompose and transform some contaminants, reduce the harmful effect of contaminants on soil and plants, and promote plant growth to a certain extent [10]. Plant roots, associated with the rhizosphere bacteria, show a significant role in detoxifying contaminants [30,41].

According to Chouychai et al. [42], corn root growth exhibited the least sensitivity to contaminants, although its germination rate was the lowest in their presence. The combined presence of both PAHs resulted in a more pronounced reduction in groundnut shoot and root lengths compared to phenanthrene or pyrene alone. Conversely, the lengths of corn shoots and roots decreased to a similar extent when either phenanthrene or pyrene was present alone. In oil-contaminated soil, corn roots grew faster than groundnut roots. In light of these findings, the authors concluded that corn is the most suitable crop for cultivation in soil contaminated with PAHs.

## 4. Materials and Methods

### 4.1. Reagents

The reagents utilized in this study included phenanthrene and naphthalene, both with a purity of 98%, obtained from the Sinopharm Chemical Reagent Co. Sigma-Aldrich (Shanghai, China). Chromatographic grade methanol and other analytically pure reagents were also employed. Corn seeds were used, specifically silage corn seeds of the “Dajingjiu 26” variety. These seeds were characterized by a germination rate of no less than 90% and a purity of no less than 96%.

### 4.2. Preparation of Bacterial Solution

Pure and complex bacteria were inoculated on Luria–Bertani (LB) nutrient medium and cultured in a rotatory shaker (BLabotery ZQPZ-228A, Tianjin, China) at 28 °C for 48 h. Following centrifugation at 8000 rpm for 4 min, the collected bacteria cells were washed with a basic inorganic salt medium (0.5 g·L^−1^ NaCl, 1.5 g·L^−1^ K_2_HPO_4_, 1.0 g·L^−1^ KH_2_PO_4_, 1.0 g·L^−1^ (NH_4_)_2_SO_4_, 0.2 g·L^−1^ MgSO_4_·7H_2_O, pH 7.0) three times. The bacterial solution was re-suspended, and the concentration was adjusted to OD600 = 1.0 (10^8^ colony forming units (CFU)·mL^−1^) for use. Prior to assessing growth through OD600 measurement, cell viability (CFU/mL) was determined via serial dilutions ranging from 10^5^ to 10^9^. The cultures were grown on LB agar, and the cell count was calculated using a calibration curve correlating OD600 values to CFU/mL. Additionally, samples were subjected to two-fold or more serial dilutions until the OD600 reading was ≤1 [19,43,44].

### 4.3. Preparation of Synthetic PAH-Contaminated Soil

The test soil was sourced from the surface layer of farmland on the outskirts of Zhenjiang City, Jiangsu Province, China, with a sampling depth of 20 cm. Following the retrieval of soil samples, removal of dead branches and leaves, and air drying, the soil was sieved through a 10-mesh sieve. To prepare synthetic PAH-contaminated soil, a standard solution of phenanthrene and naphthalene (2.5 g each, dissolved in 50 mL acetone solution) was added to achieve a final concentration of approximately 100 mg·kg^−1^. The soil was weighed into pots, and water was added after determining the concentrations of phenanthrene and naphthalene.

### 4.4. Plant Preparation

Corn seeds underwent disinfection with a 5% sodium hypochlorite solution for 5–10 min, followed by immersion in sterile water for 2 h. After promoting germination at 25 °C, the seeds were sown in seedling trays. The seedling tray was filled with a soil mixture (nutrient soil: vermiculite: perlite: volcanic rock = 5:3:1:1, volume ratio) and subjected to high-pressure steam sterilization. Germinated corn seeds were transplanted and cultured in a constant temperature plant culture chamber with a light cycle of 16 h and a chamber temperature set to 24 °C. Pot experiments were conducted on the growth of three leaves in the plants.

### 4.5. Pot Experiment Design

Four strains of domesticated bacteria were used in this study after being acclimated from polluted farmland soil near a coke plant and activated sludge from a wastewater plant. Our previous study reported on the community composition and diversity analysis of microbial strains, and six pure degrading bacteria strains were selected from them [45]. The designation of domesticated and degrading bacteria was based on their origins, including soil phenanthrene-degrading complex flora T1; soil naphthalene-degrading complex flora T2; activated sludge degradation of phenanthrene complex bacteria W1; activated sludge degradation of naphthalene complex bacteria W2; phenanthrene-degrading pure bacteria F2-6, EF3, and Nei2; and naphthalene-degrading bacteria N1-3, N2-2, and ETN2 (Table S1). These PAH-degrading bacteria strains were then grown on nutrient media before being inoculated in PAH-contaminated soil (Table S2). Inoculation involved watering the PAH-degrading bacterial solution on the 2nd day after transplanting corn seedlings and at the middle stage of culture (15th day). The quantity per pot was 30 mL, and the degrading bacteria had OD600 = 1.0.

Experimental groups were designated as follows: CK (contaminated soil), P (contaminated soil + plants), CP (uncontaminated soil + plants), B (contaminated soil + degrading bacteria), and PB (contaminated soil + degrading bacteria + plants). Each group had three replicates. Regular watering was carried out to maintain a water content of approximately 60%, with basin exchange occurring every two days. The temperature was maintained at 25 °C, and the light cycle was set to 16 h of light/8 h of darkness. Four domesticated bacteria strains were used in this study following acclimation from polluted farmland soil near a coke plant and activated sludge from a wastewater plant. At the same time, the community composition and diversity analysis of microbial strains were reported in our previous study [45], and six pure bacteria strains were selected from them. The designation of domesticated and degrading bacteria was based on their origins, including soil phenanthrene-degrading complex flora T1; soil naphthalene-degrading complex flora T2; activated sludge degradation of phenanthrene complex bacteria W1; activated sludge degradation of naphthalene complex bacteria W2; phenanthrene-degrading pure bacteria F2-6, EF3, and Nei2; and naphthalene-degrading bacteria N1-3, N2-2, and ETN2 (Table S1). These PAH-degrading bacteria strains were subsequently grown on nutrient media before inoculation in PAH-contaminated soil (Table S2).

### 4.6. Determination of Soil pH

Soil samples from each pot were mixed after 5 days, 10 days, and 30 days of the experiment. Then, 10 g of soil were weighed, and 50 mL of sterile water was added. After shaking on a 180 rpm rotatory shaker for 2 h, the pH of the soil solution was measured using a rapid LAQUAtwin Compact pH meter (ASpH22, Horiba Advanced Techno Co., Ltd., Kyoto, Japan).

### 4.7. Bacteria Counting in Soil

To assess the bacteria count in the soil, a specific amount of dry potting soil was weighed in mortar, ground, and thoroughly mixed. Subsequently, 10 g of the soil sample was weighed in a conical flask, and 20 mL of aseptic water was added. The flask underwent shaking in a rotatory shaker for 2 h, followed by a 30 min settling period. The supernatant was then diluted into 10^−1^, 10^−2^, 10^−3^, and 10^−4^ concentrations. These dilutions were applied in 50 µL portions on LB media plates and incubated at 28 °C for 48 h. Colony counting was performed, and the number of bacteria per unit of soil mass was calculated based on dilution. Each experiment was carried out in triplicate, and the CFU of microorganisms was log-transformed.

### 4.8. Determination of Phenanthrene and Naphthalene Concentrations in Soil

Silica gel and anhydrous sodium sulfate were activated before use. Firstly, a 10 g soil sample was weighed into a tray, and 20 mL of dichloromethane was added. After stirring to eliminate bubbles in the silica gel, the mixture was drained into a chromatographic column. The chromatographic column was rinsed with dichloromethane, and the eluent was collected. Subsequent steps involved washing with n-hexane, adding anhydrous sodium sulfate, and rinsing with dichloromethane and n-hexane solution. The sample solution was transferred to the chromatographic column, and the eluent was collected. The eluent was analyzed using high-performance liquid chromatography (HPLC) (Prominence LC, Shimadzu Co., Kyoto, Japan). The HPLC parameters were set as follows: a C18 reversed-phase column (4.6 mm × 250 mm) was employed with a mobile phase consisting of methanol and water (90:10); detection occurred at 254 nm. The flow rate was maintained at 1.0 mL · min^−1^, and the column temperature was set to 40 °C. Each sample, with an injection volume of 20 μL, underwent a 25 min run time. All experiments were conducted in triplicate. Initial concentrations of phenanthrene and naphthalene in soil were calculated based on the absorbance area and standard curve. The removal rate of phenanthrene and naphthalene in soil was calculated according to Equation (1):

(1) $$R=\frac{Co-Ct}{Co}\times 100\%$$

where R is the removal rate of the PAHs, *Co* is the initial concentration of PAHs in the soil, and *Ct* is the concentration of PAHs at culture time *t*.

### 4.9. Determination of Phenanthrene and Naphthalene in Corn Plants

During the investigation of phenanthrene and naphthalene levels within corn plants, the plant samples underwent cutting and mixing, and subsequently, a specific quantity was placed into a 25 mL centrifuge tube. A solution of 10 mL acetone and n-hexane with a volume ratio of 1:1 was introduced into the centrifuge tube. Ultrasonic extraction was then conducted in a water bath for 30 min. The aforementioned steps were repeated twice, and the resultant extraction solution was collected and passed through an anhydrous sodium sulfate column, transferred to a rotary evaporation bottle, and concentrated to dryness at a consistent temperature of 40 °C. The volume was adjusted to 2 mL using n-hexane. A 1 mL solution was purified through a silica gel column and eluted with a 1:1 dichloromethane and n-hexane volume ratio. The eluent was collected in a colorimetric dish and dried with nitrogen at room temperature. The methanol volume was standardized to 2 mL, and the methanol passed through a 0.22 μm organic phase filter membrane before being analyzed using HPLC as previously described. The initial concentration of phenanthrene and naphthalene in plant tissues was determined based on the standard curve derived from absorbance area measurements. The translocation factor (TF) of corn to phenanthrene or naphthalene was calculated according to Equation (2):

(2) $$\mathrm{TF}=\frac{\mathrm{concentration in the aboveground part of the plant}}{\mathrm{concentration in the underground part}}$$

### 4.10. Determination of Corn Biomass Yield

After 30 days of potted plant growth, the fresh weight of the shoots and root parts was measured. After drying at 65 °C to a constant weight, the dry weight was determined, and the water content was calculated.

### 4.11. Statistical Analysis

Microsoft Office Excel 2016 software (64-Bit Edition)was employed in data processing, and the outcomes were presented as “mean ± standard deviation”. The one-way ANOVA analysis utilized SPSS Statistics 25.0 software, employing the least significant difference method for assessing significance. However, once homogeneity among variables was confirmed, treatment level means were paired using Tukey’s Honest Significant Difference (HSD) test at a significance level of *p* < 0.05. The graphical representations essential for the study were also created using Origin 2018.

## 5. Conclusions

This study assessed the removal rates of phenanthrene and naphthalene using PAHs-degrading bacteria, corn, or their consortia, aiming to accelerate the removal of these contaminants from both soil and corn plants. The results underscore the significant role of the corn and degrading bacteria consortium in reducing PAH contamination in soil. Specifically, the consortium led to a significant (*p* < 0.05) increase in the bacterial population in phenanthrene- and naphthalene-contaminated soil. Furthermore, the corn and degrading bacteria consortium did not result in a significant accumulation of phenanthrene or naphthalene in corn plants. Moreover, the corn biomass yield from all combined corn and degrading bacteria remediation methods was significantly higher compared to the single-plant remediation approach. These findings suggest that incorporating degrading bacteria within the corn and degrading bacteria consortium holds promise for alleviating the toxic effects of phenanthrene or naphthalene on corn plants, thereby enhancing the corn biomass yield.

## Figures and Tables

**Table 1:** Effects of remediation methods on phenanthrene and naphthalene accumulation in corn.

PAH	Remediation Method	Shoot Accumulation (mg·kg^−1^)	Root Accumulation (mg·kg^−1^)	Translocation Factor (TF)
Phenanthrene	P	0.063 ± 0.001 ^a^	0.215 ± 0.011 ^a^	0.296 ± 0.171 ^a^
	PB-T1	0.101 ± 0.006 ^c^	0.308 ± 0.007 ^c^	0.329 ± 0.273 ^a^
	PB-W1	0.131 ± 0.007 ^d^	0.380 ± 0.008 ^cd^	0.348 ± 0.139 ^a^
	PB-EF3	0.093 ± 0.001 ^bc^	0.290 ± 0.006 ^b^	0.322 ± 0.212 ^a^
	PB-Nei2	0.086 ± 0.002 ^b^	0.296 ± 0.004 ^bc^	0.294 ± 0.350 ^a^
	PB-F2-6	0.096 ± 0.003 ^bc^	0.304 ± 0.008 ^bc^	0.316 ± 0.431 ^a^
Naphthalene	P	0.103 ± 0.022 ^a^	0.379 ± 0.008 ^a^	0.274 ± 0.064 ^a^
	PB-T2	0.141 ± 0.013 ^a^	0.413 ± 0.010 ^a^	0.343 ± 0.034 ^a^
	PB-W2	0.153 ± 0.019 ^a^	0.427 ± 0.015 ^a^	0.358 ± 0.041 ^a^
	PB-N1-3	0.112 ± 0.010 ^a^	0.343 ± 0.013 ^a^	0.311 ± 0.034 ^a^
	PB-N2-2	0.106 ± 0.017 ^a^	0.373 ± 0.019 ^a^	0.311 ± 0.066 ^a^
	PB-ETN2	0.110 ± 0.011 ^a^	0.382 ± 0.009 ^a^	0.296 ± 0.024 ^a^

Different superscript letters indicate significant differences in phenanthrene and naphthalene contents in corn tissues between different remediation methods (*p* < 0.05).

**Table 2:** Effects of phenanthrene- and naphthalene-degrading bacteria on biomass yield of corn.

Pah	Remediation Method	Shoot Biomass		Root Biomass	
		Fresh Weight (g)	Water Content (%)	Fresh Weight (g)	Water Content (%)
	CP	5.19 ± 0.43 ^a^	81.46	2.30 ± 0.20 ^ab^	75.02
Phenanthrene	P	4.16 ± 0.45 ^b^	82.94	1.78 ± 0.12 ^c^	73.60
	PB-T1	5.15 ± 0.42 ^a^	82.92	2.05 ± 0.18 ^bc^	71.71
	PB-W1	5.24 ± 0.52 ^a^	82.45	2.40 ± 0.19 ^ab^	70.42
	PB-EF3	4.97 ± 0.31 ^ab^	83.71	2.12 ± 0.28 ^bc^	75.48
	PB-Nei2	4.27 ± 0.40 ^b^	82.21	2.22 ± 0.19 ^ab^	77.93
	PB-F2-6	4.54 ± 0.47 ^ab^	82.60	2.54 ± 0.14 ^a^	78.35
	CP	5.19 ± 0.43 ^ab^	81.46	2.30 ± 0.20 ^a^	75.02
Naphthalene	P	3.80 ± 0.37 ^c^	82.85	1.76 ± 0.26 ^c^	77.28
	PB-T2	4.87 ± 0.31 ^b^	80.01	1.86 ± 0.23 ^bc^	72.58
	PB-W2	5.61 ± 0.40 ^a^	82.00	2.20 ± 0.16 ^ab^	72.28
	PB-N1-3	5.05 ± 0.37 ^ab^	83.17	1.96 ± 0.20 ^abc^	76.54
	PB-N2-2	4.66 ± 0.30 ^b^	82.62	1.68 ± 0.14 ^c^	73.22
	PB-ETN2	4.57 ± 0.35 ^b^	83.37	1.79 ± 0.22 ^bc^	76.98

Different superscript letters indicate significant differences in corn biomass yield between different remediation methods (*p* < 0.05).

## Data Availability

Data will be made available on request.

## Supplementary Materials

The following supporting information can be downloaded at: https://www.mdpi.com/article/10.3390/plants13202839/s1, Table S1: Domesticated bacterial flora and degrading bacterial strains in this study; Table S2: Degradation efficiency of the domesticated bacterial flora and degrading bacterial strains in nutrient media; Table S3: pH of soil contaminated by different phenanthrene-and naphthalene-degrading bacteria; Table S4: Number of bacteria in potting soil under different remediation methods.
