# Supplementary material for: Enhanced Remediation of Phenanthrene and Naphthalene by Corn-Bacterial Consortium in Contaminated Soil
Source: Plants (Basel). 2024 Oct 10;13(20):2839. doi: 10.3390/plants13202839 (PMC11511142; doi:10.3390/plants13202839)
Supplement: Supplementary file 1 [file plants-13-02839-s001.zip › plants-3197375-supplementary.pdf]

Supplementary information

Remediation of phenanthrene and naphthalene by corn-bacterial consortium in contaminated soil

Lu GAO [a1], Charles Obinwanne OKOYE [a, b, c1], Congsheng WANG [a], Feiyue LOU [a], Jianxiong JIANG [a*]

[a] *Biofuels Institute, School of Environment & Safety Engineering, Jiangsu University, Zhenjiang 212013, China*

[b] *School of Life Sciences, Jiangsu University, Zhenjiang 212013, China*

[c] *Department of Zoology & Environmental Biology, University of Nigeria, Nsukka 410001, Nigeria*

* Corresponding author at: Biofuels Institute, Jiangsu University, No. 301 Xuefu Road, Zhenjiang 212013, China.

E-mail address: jxjiang@ujs.edu.cn; Tel: +86-511-8879-6122 (J. Jiang).

[1] Shared the first author.

Table S1. Domesticated bacterial flora and degrading bacterial strains in this study.

Substrate for domestication or screening	Numbers	Origin
Phenanthrene	T1	Soil degrading complex flora
	W1	Activated sludge degradation complex bacteria
	Nei2	*Comamonas pittii*
	EF3	*Klebsiella oxytoca*
	F2-6	*Arthrobacter oxydans*
Naphthalene	T2	Soil degrading complex flora
	W2	Activated sludge degradation complex bacteria
	N1-3	*Pseudomonas putida*
	N2-2	*Comamonas aquatica*
	ETN2	*Klebsiella pneumoniae*

Table S2. Degradation efficiency of the domesticated bacterial flora and degrading bacterial strains in nutrient media.

PAH	Numbers	Degradation efficiency (%)
Phenanthrene	T1	52.63 ± 5.97^{c}
	W1	85.89 ± 4.80^{a}
	EF3	77.71 ± 1.78^{b}
	Nei2	41.22 ± 1.00^{d}
	F2-6	47.27 ± 3.56^{cd}
Naphthalene	T2	52.15 ± 10.7^{bc}
	W2	79.45 ± 6.67^{a}
	N1-3	64.61 ± 5.80^{b}
	N2-2	48.60 ± 4.55^{c}
	ETN2	42.54 ± 5.42^{c}

Table S3. pH of soil contaminated by different phenanthrene-and naphthalene-degrading bacteria.

Remediation method	Phenanthrene-contaminated soil pH			Remediation method	Naphthalene-contaminated soil pH		
	5d	10d	30d		5d	10d	30d
CK	7.20[a]	7.23[a]	7.26[a]	CK	7.20[a]	7.18[a]	7.19[a]
P	7.26[a]	7.39[a]	7.73[a]	P	7.28[a]	7.42[a]	7.63[a]
B-T1	7.25[a]	7.28[a]	7.42[a]	B-T2	7.21[a]	7.28[a]	7.40[a]
B-W1	7.25[a]	7.30[a]	7.35[a]	B-W2	7.14[a]	7.17[a]	7.35[a]
B-EF3	7.21[a]	7.29[a]	7.44[a]	B-N1-3	7.19[a]	7.20[a]	7.33[a]
B-Nei2	7.24[a]	7.35[a]	7.52[a]	B-N2-2	7.18[a]	7.25[a]	7.39[a]
B-F2-6	7.23[a]	7.38[a]	7.50[a]	B-ETN2	7.17[a]	7.29[a]	7.32[a]
PB-T1	7.25[a]	7.42[a]	7.65[a]	PB-T2	7.31[a]	7.49[a]	7.69[a]
PB-W1	7.27[a]	7.33[a]	7.60[a]	PB-W2	7.28[a]	7.45[a]	7.63[a]
PB-EF3	7.26[a]	7.30[a]	7.77[a]	PB-N1-3	7.32[a]	7.48[a]	7.59[a]
PB-Nei2	7.27[a]	7.29[a]	7.37[a]	PB-N2-2	7.27[a]	7.47[a]	7.68[a]
P-F2-6	7.28[a]	7.28[a]	7.72[a]	PB-ETN2	7.25[d]	7.42[a]	7.71[a]

Different superscript letters indicate significant differences in soil pH between different remediation methods (p<0.05).

This study determined the pH value of rhizosphere soil in different remediation methods after contamination with phenanthrene and naphthalene. The results showed that with the extension of the test time, the soil pH value slightly increased but was not significant (p>0.05) among the different remediation methods and CK (**Table S3**).

After 30 days, PB-EF3 (7.77) and PB-ETN2 (7.71) were highest, although not significantly different from the other remediation methods for phenanthrene- and naphthalene-contaminated soils.

Table S4. Number of bacteria in rhizosphere soil under different remediation methods.

Remediation method	Number of bacteria (10^8 CFU·g^{-1})			Remediation method	Number of bacteria (10^8 CFU·g^{-1})		
	5d	10d	30d		5d	10d	30d
CK	2^{a}	2^{a}	3^{a}	CK	3^{a}	3^{a}	4^{a}
P	3^{a}	4^{a}	6^{ab}	P	3^{a}	4^{a}	6^{a}
B-T1	30^{c}	22^{b}	18^{e}	B-T2	32^{cd}	26^{c}	14^{d}
B-W1	27^{b}	21^{b}	12^{d}	B-W2	35^{d}	29^{d}	19^{e}
B-EF3	33^{d}	24^{c}	9^{c}	B-N1-3	27^{b}	19^{b}	9^{c}
B-Nei2	29^{bc}	22^{b}	7^{b}	B-N2-2	29^{bc}	27^{c}	7^{b}
B-F2-6	28^{bc}	23^{bc}	8^{bc}	B-ETN2	30^{c}	26^{c}	7^{b}
PB-T1	35^{de}	54^{e}	75^{fg}	PB-T2	33^{cd}	65^{g}	78^{h}
PB-W1	29^{bc}	49^{d}	83^{h}	PB-W2	37^{de}	73^{h}	82^{i}
PB-EF3	27^{b}	52^{e}	79^{g}	PB-N1-3	39^{e}	66^{g}	76^{g}
PB-Nei2	32^{d}	63^{f}	78^{g}	PB-N2-2	32^{cd}	59^{f}	74^{g}
PB-F2-6	25^{b}	64^{f}	72^{f}	PB-ETN2	28^{b}	54^{e}	71^{f}

Different superscript letters indicate significant differences in bacteria numbers between different remediation methods (p<0.05).

The number of corn rhizosphere bacteria was counted to reveal the effect of corn and degrading bacteria or their combination on the proliferation of bacteria in soil (**Table S4**). The results showed a consistent trend in the number of bacteria in the soil. Compared to CK, the contaminated soil treated with degrading bacteria showed a

gradually decreasing trend in the number of bacteria with time. On the other hand, in all the soils treated with the PB remediation methods, the number of soil bacteria increased to 2-3 times that on the 5th day, with PB-W1 (83×10^8 CFU·g^{-1}) and PB-W2 (82×10^8 CFU·g^{-1}) treatment methods have the highest ($p<0.05$) numbers of rhizosphere bacteria after 30 days.
